# Ripples in the pond: Evidence for contagious cooperative role modeling through moral elevation and calling in a small pre-study

**DOI:** 10.3389/fpsyg.2022.1005772

**Published:** 2022-10-20

**Authors:** Qionghan Zhang, Jianhong Ma, Yuqi Wang, Xiqian Lu, Changcun Fan

**Affiliations:** ^1^School of Business Administration, Zhejiang Gongshang University, Hangzhou, China; ^2^Department of Psychology and Behavioral Sciences, Zhejiang University, Hangzhou, China; ^3^Institute of Psychology, Chinese Academy of Sciences, Beijing, China

**Keywords:** prosocial modeling, cooperation, public goods, goal contagion, moral elevation, calling

## Abstract

Existing research has identified the importance of role models in the imitation of cooperative behaviors. This Pre-Study attempted to explore the contagion effects of cooperative models. Drawing on goal contagion theory, we proposed that encountering cooperative models could catalyze participants’ cooperation when participants joined new groups without role models, and that moral elevation and calling would play a chain-mediating role in this process. To test the hypothesis, we designed a four-person public goods game consisting of two phases in which participants were formed into teams with different people in each phase. We randomly assigned 108 participants to either a consistent contributor (CC) or control condition. The only difference was that participants in the CC condition encountered a cooperative role model (i.e., CC) in the first phase, while those in the control group did not. The results moderately supported all hypotheses. Briefly, our findings provide empirical evidence supporting the two processes of goal contagion theory: when individuals encounter a CC, they first make inferences about the CC’s goal, as reflected by moral elevation, and then adopt the model’s prosocial goals (i.e., calling), resulting in increased cooperative behaviors in new groups. These findings could extend our understanding of the contagion effect of cooperative modeling, but require high-powered replication studies before such conclusions can be drawn.

## Introduction

The sustainability of cooperation remains a critical issue in various fields, such as in coping with the COVID-19 pandemic and environmental protection. In responding to the COVID-19 crisis, identifying potential threats, sharing critical information, complying with safety guidelines, and adopting preventative behaviors all require cooperation at the government and individual levels (Capraro et al., 2021; Yong and Choy, 2021). In addition, environmental protection requires cooperation not only among different countries but also across generations (Van Lange and Rand, 2022). Thus, understanding human cooperation is necessary for dealing with these issues.

The traditional rational choice theory suggests that maintaining cooperation through voluntary contributions is not sustainable (Andreoni, 1988; Fehr and Schmidt, 1999). Further, when studying cooperation, researchers have often made examinations at the micro level within the context of social dilemma situations comprising conflicts between private and collective interests (Dawes, 1980; Fleishman, 1988; Yuan et al., 2022). Specifically, in a social dilemma, individuals who choose not to cooperate always gain greater benefits than cooperators, whereas everyone benefits more when everyone cooperates than when everyone does not cooperate (Dawes, 1980; Chen, 2022). In such a context, the presence of a free rider—namely, an individual or group of individuals who benefit from a group endeavor to which they did not contribute—could easily destabilize group cooperation (Andreoni, 1988; Panchanathan and Boyd, 2004; Naso, 2020). Consequently, researchers are interested in solutions that can improve or spread cooperation. Some academics believe that structural solutions, such as sanctions and rewards, can be beneficial in maintaining cooperation (Fehr and Gächter, 2002; De Quervain et al., 2004; Fehr and Fischbacher, 2004; Kiyonari and Barclay, 2008). However, a growing body of evidence demonstrates that such solutions are typically more resource-intensive, diminish individuals’ inner motivation to cooperate, and sanctions can attract a vicious cycle of retaliation(Deci et al., 1999; Mulder et al., 2006; Dreber et al., 2008; Herrmann et al., 2008). Then, due to their low cost and capacity to alter people’s perceptions of the extrinsic environment, motivational solutions are gaining traction among academicians (Van Lange et al., 2013; Iwai and de Azevedo, 2016; Zhang et al., 2019).

One typical example of such motivational solutions is role modeling, which has recently been identified as crucial to the emergence, development, and establishment of cooperation (House et al., 2020; Jung et al., 2020). According to the culture–gene coevolution theory, the greatest difference between humans and other species is that humans, as a cultural species, rely heavily on the vast amount of social knowledge they have collected over generations (Chudek et al., 2013). Further, humans can obtain social knowledge through direct experience, inheritance from parents (vertical genetic transmission), and learning from non-parental role models (horizontal cultural transmission) (Creanza et al., 2017). Among the various types of social knowledge, cooperation is significant because it can assist groups, unions, or even societies in coping with competition and dangers in nature (Nowak and Highfield, 2011; Francois et al., 2018). Apart from that, researchers have claimed that humans biologically evolved for cooperation due to having a unique motivation to share their understanding of the goals, intentions, and perceptions of others, as well as certain forms of cognitive representation for doing so (Tomasello et al., 2005). Consequently, cooperation has become an evolutionary superior strategy that is widely acquired and transmitted through dual genetic and cultural inheritance systems (Henrich and Muthukrishna, 2021).

Empirical studies have found converging evidence regarding the effects of modeling on cooperation, and one of the most typical effects is the consistent contributor (CC) effect. In the public goods dilemma, one of the classic and widely used social dilemmas to investigate group cooperation, Weber and Murnighan (2008) reported a CC effect whereby individuals observed a group member who consistently contributed own endowment to the public account (i.e., a manifestation of very determined cooperative behavior); this observation then led individuals to follow the role model and increase their cooperative behaviors.

Previous studies have generally focused on participants’ cooperative behaviors in the presence of CCs (Gill et al., 2013; Zhang et al., 2019); however, only a few research have examined participants’ cooperative behaviors after they left the environment with the CCs and entered a new environment (Suri and Watts, 2011). As a matter of fact, instant imitation is simply the starting point for the cooperative modeling effect. It is vital that individuals continue to demonstrate cooperative behaviors outside the group or context in which the role model (e.g., a CC) performs such behaviors. This is because, if an individual’s cooperative behavior is limited to the environment in which the role model is present, the cooperative model can only impact the groups to which the role model is exposed. Conversely, if individuals can acquire the role models’ cooperative behaviors and maintain them upon entering a new environment, they may become “cooperative models” in the new setting or group. Through this contagion effect, the influence of a single cooperative model can be transmitted to a large number of people, just like “ripples in a pond.” Furthermore, examinations to clarify the breadth of the effect of cooperative role models can improve our understanding of culture–gene coevolution theory. Therefore, this study sought to determine whether there is a CC contagion effect and its potential underlying mechanisms.

### CC effect

The CC effect refers to the phenomenon of increased cooperative behavior in group members induced by a CC, as observed in a public goods dilemma (Weber and Murnighan, 2008; Zhang et al., 2019). In a classic all-or-none public goods game, a group of individuals will each receive a certain number of tokens, and they must each choose whether to contribute these tokens to a public account or their personal accounts. Individuals receive a set amount of dividends from the public account regardless of their contribution to it. Consequently, not contributing to the public account (i.e., selfish behavior) is typically considered a rational strategy in such a dilemma, while contributing to the public account (i.e., cooperative behavior) is thought highly by people. The existence of the CC effect has been confirmed in many variants of public goods games, such as all-or-none, continuous, and step-level public goods games (Weber and Murnighan, 2008; Gill et al., 2013; Zhang et al., 2019).

Several divergent accounts for explaining the CC effect have been proposed. Some scholars have explained this phenomenon using social norms. Using the “logic of appropriateness framework” to explain the CC effect, academicians claimed that the presence of a CC sends a clear signal to the group members that cooperation is appropriate behavior in the present context, implying that cooperation is the group norm (Weber et al., 2004; Weber and Murnighan, 2008). In the minority influence framework, researchers added that by consistently modeling cooperative behavior, minority individuals are able to challenge accepted norms of self-interest and transition them to cooperative norms (Grant and Patil, 2012). Other scientists have corroborated the mediating role of moral elevation—which is defined as an emotional experience of a warm and uplifting feeling experienced when individuals see unanticipated acts of kindness by other persons (Haidt, 2000), on the CC effect (Gill et al., 2013; Zhang et al., 2019; Huang et al., 2022).

### The contagion of cooperative modeling

Researchers have described the contagion of prosocial modeling in the form of Person A-B-C as generalized reciprocity (Tsvetkova and Macy, 2014), upstream reciprocity (Norbutas and Corten, 2018), or pay-it-forward (Gray et al., 2014). While extensive literature has provided consistent evidence regarding the contagion effect of helping modeling behaviors (Gray et al., 2014; Tsvetkova and Macy, 2014; Alvarez and van Leeuwen, 2015; Chancellor et al., 2018; Eriksson and Ferreira, 2021), there is still uncertainty regarding the contagion effect of cooperative modeling.

Some studies have indicated that when individuals experience cooperative modeling behaviors, they also perform cooperative behaviors upon entering new settings, thus demonstrating the contagion effect of cooperative modeling. For example, a study reported that cooperative behavior could cascade in human social networks, continue over time, and extend up to three degrees of separation (Fowler and Christakis, 2010). Another study discovered that group leaders who have the power to punish team members also have a contagious impact as cooperative role models, meaning that their cooperative behavior affects group members’ cooperative behavior both inside and outside the group (Harrell, 2019).

However, not all studies have found favorable results regarding the contagion effect of cooperative modeling behaviors. While Suri and Watts (2011) confirmed the CC effect in a web-based, networked public goods game, they did not find evidence for the CC contagion effect. Their interpretation of this was that the presence of CCs might encourage free riding. Similarly, Jordan et al. (2013) reported that cooperative modeling behavior is infectious in a relatively fixed group, but not viral in more dynamic networks. This was measured by moving participants into different groups after each round of a public goods game.

Consequently, this study investigated the contagion effect of CCs. In a recent detailed meta-analytic review of prosocial modeling, Jung et al. (2020) compared four frameworks that might explain the psychological mechanisms of prosocial role models: behavioral mimicry, goal contagion, situational pressure, and experimenter effect. The result supports the goal contagion theory, which contends that prosocial role models motivate other individuals to mimic their behavior by adopting similar prosocial goals (Aarts et al., 2004; Corcoran et al., 2020). Although this meta-analysis did not include a series of publications on CC, it is still quite instructive. Motivated by goal contagion theory, we contend that group members who experience a CC acquire the prosocial goals of the CC, exhibiting cooperative behavior as a result, even when joining a new/different group or context.

### The chain-mediating role of moral elevation and calling

#### The goal contagion theory

The goal contagion theory claims that when individuals observe or experience others’ behavior, they infer the goal of the other’s behavior and may decide to adopt the goal (Aarts et al., 2004; Corcoran et al., 2020). Goal contagion is typically viewed as a two-stage process, inferring the goal of the role model and adopting that goal (Brohmer et al., 2019; Corcoran et al., 2020). Goal inference includes an explicit conscious component and may also include implicit unconscious automatic processing (Dik and Aarts, 2007; Corcoran et al., 2020). Because implicit and explicit goal inference may coexist, researchers have encountered many problems with the measurement and validation of goal inference. First, explicit measures of goal inference can easily interfere with automatic processes, while some measures of implicit goal inference may prime some constructs related to the goal (Weingarten et al., 2016; Corcoran et al., 2020). Second, uniform standards for the measurement of goal inference are currently lacking, and many assessments often confound some other factors, such as goal adoption (Jia et al., 2014).

Under these conditions, the empirical validation of the mediating role of goal inference in goal contagion theory is extremely limited. For example, Dik and Aarts (2007) measured explicit goal inference in an experiment but did not find a mediating role in the goal contagion effect. Corcoran et al. (2020) tried to disentangle explicit and implicit inference as potential mediators in the goal contagion effect, but did not find evidence for either process. Meanwhile, goal adoption is frequently assessed by an individual’s goal-directed behavior or their inclination to engage in it (Brohmer et al., 2021), and seldom have researchers found unambiguous evidence in support of a specific goal adoption process.

Due to the difficulties in measuring this two-stage process, few studies have empirically examined them separately, not to mention in the context of cooperative modeling. Consequently, this study attempts to separately validate the two processes of goal contagion theory to provide insights into the mechanisms underlying the CC contagion effect. To achieve this objective, we innovatively introduced moral elevation and calling as proxy variables for goal inference and goal adoption.

#### Moral elevation

As aforementioned, moral elevation refers to a warm and uplifting feeling related to unanticipated kindness by others (Haidt, 2000), and scholars have stated that it triggers with the attention to and the observation and praise of others’ moral behavior of others, therefore describing it as a positive other-praising emotion (Thomson and Siegel, 2017). It is also described as involving feelings of self-transcendence (Haidt and Morris, 2009; Van Cappellen et al., 2013). Researchers have already confirmed the full mediating role of moral elevation in the positive relationship between CC presence and group members’ cooperative behaviors (Zhang et al., 2019). However, we expect it to also play a vital role in the CC contagion effect, as followers.

First, a key prerequisite for moral elevation is that individuals need to develop positive appraisals of the moral behavior of others and attribute the cause of the behavior to others’ virtue. For example, Van de Vyver and Abrams (2015) demonstrated that positive evaluations of others’ moral virtue mediated the generation of individuals’ elevation after they viewed videos of others’ moral behavior. Similarly, Ash (2017) confirmed that those who watched a black-savior-themed movie experienced moral elevation through a positive assessment of the savior’s morality. Put differently, moral elevation is the emotional reaction of a person who has completed a goal inference regarding a role model and confirms the prosocial nature of that goal. Another study, wherein participants were asked to read a story and evaluate whether the leader in the story sacrificed himself to help the company, shows that the participants’ positive perception of the leader’s behavior and goal induced the participants’ positive evaluation of own job by stimulating moral elevation. Thus, we argue that individuals’ moral elevation because of observing others’ virtuous behaviors is a reflection of individuals’ explicit inference about others’ prosocial goals. Since goal inference is difficult to explicitly assess, moral elevation could be a superior alternative measurement (Vianello et al., 2010).

In addition, moral elevation can boost people’s prosocial motivations and behaviors (Ding et al., 2018; Rullo et al., 2022). Algoe and Haidt (2009) discovered that participants who were aroused to a high level of moral elevation after watching a role model video reported: motivation to emulate the role model’s behavior; prosocial motivation; the possibility of acting on these motivations. Another study found that participants were more eager to take part in further unpaid research and invest time and energy to help researchers with additional tasks when they were inspired by a high level of moral elevation due to watching videos of others’ positive ethical behavior (Schnall et al., 2010).

As such, it would be reasonable to expect that participants in CC groups may attribute CCs’ behavior to the CCs’ good qualities, thus generating a feeling of moral elevation, which further influences their cooperative behavior beyond CC’s setting.

#### Calling

As explicitly evaluating the process through which individuals adopt the prosocial goals of CCs is problematic, we can measure individuals’ prosocial goals after they encounter CCs. An example of a prosocial goal closely related to this topic is calling, referring to the goals of reaching beyond self-actualization and achieving a higher purpose for the greater good (Wong, 2013). Dik and Duffy (2009) defined this prosocial goal as a transcendent summons that is felt as coming from outside oneself, directing one to undertake a certain life role that emphasizes other-oriented values and goals as the main sources of motivation. Researchers show that Maslow further posited, in his new hierarchy of needs, that certain self-actualized individuals could be inspired to commit to the fulfillment of callings beyond themselves to reach a higher level of self-transcendence (Koltko-Rivera, 2006). Researchers have also described a calling as a sense of purpose, often directed outside of oneself in an altruistic manner (Selvam and Poulsom, 2012). Following a thorough examination of the prior literature, Elangovan et al. (2010) stressed three key characteristics of calling that have persisted throughout the numerous ways interpretations of the term: action orientation, a sense of clarity of purpose and personal mission, and prosocial intentions. Consequently, they defined calling as a path of activity that pursues prosocial goals and expresses the convergence of a person’s perception of what one wants to, and should do, and what one really does.

Of the three aforementioned core characteristics of calling, the prosocial intention has received ample support. For instance, a study focusing on zookeepers indicated that employees with a high level of calling exhibit more willingness to sacrifice their free time (non-work time) for their organizations (Bunderson and Thompson, 2009). Additionally, calling has been demonstrated to increase prosocial motivation in employees, which promotes green employee behavior (Zhang et al., 2021). In general, when someone expresses a sense of calling, it suggests that the person consciously identifies with one’s prosocial goal. Further, when individuals get in contact with CCs, their inferences about the prosocial goals of CCs may lead to moral elevation, an emotion of self-transcendence (Haidt and Morris, 2009; Van Cappellen et al., 2013), which is also the source from which calling stems (Dik and Duffy, 2009). Thus, it seems reasonable to assume that the generation of moral elevation might lead to the feeling of calling. As such, we believe that the consideration of calling as a proxy variable for individuals’ goal adoption after encountering a CC is appropriate, especially given its association with moral elevation.

### The current study

The first goal of the current study was to investigate the contagion effect of CC. According to the goal contagion theory, we argue that individuals who encounter a CC within a group do not simply imitate its cooperative behavior (CC effect), but rather adopt the CC’s prosocial goal and thus still exhibit cooperative behavior when entering a completely new group without the CC. Consequently, we propose the first hypothesis (H1):

*H1*: Participants in the CC group will make more cooperative decisions than those in the control group after leaving their group and entering a new group.

This research also aims to examine the appropriateness of goal contagion theory in explaining the contagion effect of CC. This theory indicates that individuals internalize role models’ goals after inferring the goals underlying their behaviors, and then act on these goals. In the situation of encountering CCs, their behaviors are typically benevolent and are often perceived to be driven by prosocial goals. By considering the difficulties experienced by past researchers in directly measuring goal inference and goal adoption in the goal contagion process, this study innovatively proposes the use of moral elevation and calling as proxy variables for goal inference and goal adoption, respectively. Accordingly, we assume that after witnessing CC’s cooperative behavior, individuals might attribute it to CC’s virtues and characteristics, generating moral elevation and thus further driving individuals to adopt CC’s prosocial goals, namely, to develop a sense of calling; this ultimately results in individuals performing cooperative behaviors in a new and different group without a CC.

To separately verify the roles of goal inference and goal adoption in the contagion effect of CC, we test the mediation hypotheses involving moral elevation and calling independently, leading to hypotheses 2 (H2) and 3 (H3):

*H2*: Moral elevation mediates the influence of CCs on participants’ subsequent cooperative decisions after participants leave their group and enter a new group.

*H3*: Calling mediates the influence of CCs on participants’ subsequent cooperative decisions after participants leave their group and enter a new group.

According to these arguments, we then propose hypothesis 4 (H4) regarding the chain-mediating role of moral elevation and calling in the CC contagion effect.

*H4*: Moral elevation and calling have a chain-mediating effect on the relationship between CCs and participants’ subsequent cooperative decisions after participants leave their group and enter a new group.

**Figure 1:**
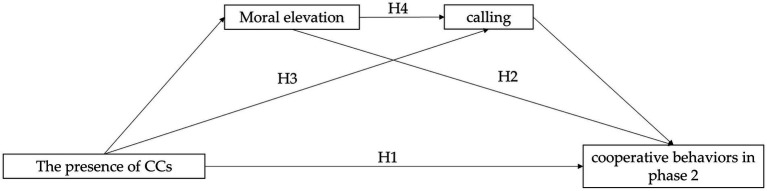
The hypothesized framework of this study.

All hypotheses are presented in Figure 1.

## Materials and methods

### Participants and design

A hundred and twelve students from Zhejiang University were recruited through the university’s online message board. Four participants were excluded because they either did not pass a test assessing the accuracy of their knowledge of the experimental rules, which was conducted after the experimental assistant explained the rules, or indicated confusion about the experimental rules at the end. The final sample comprised 108 participants (44 men and 64 women). Their average age was 21.48 years (*standard deviation* [*SD*] = 2.30).

The study had two conditions. Participants were randomly assigned to either the CC condition (*n* = 60) or the control condition (*n* = 48). Each condition comprised two phases. In the first phase, participants were formed into groups of four and had to play 15 rounds of an all-or-none public goods game. In the second phase, participants were moved to brand-new 4-person groups and played another 15 rounds of the same game in the new group. To control for the effects of social norms on individuals’ cooperative behavior (Farrow et al., 2017), the three other group members (including the CC) who interacted with participants in both phases were computer-manipulated confederates with an average likelihood of cooperative behavior of 66.7% (Gill et al., 2013). The difference between the CC and control conditions was in the first phase, where one of the simulated team members in the CC condition was a CC that consistently made cooperative decisions (i.e., contributing all the tokens to the group account); the control condition contained no CCs. In the second phase, both conditions were identical.

Incentives in public goods games are often provided to imitate real-world dilemmas in which people face conflicts between their self-interests and group interests (Goeschl et al., 2020). As such, all participants were compensated with 15–20 RMB (approximately US$2.2–3.0), depending on the number of tokens they earned during the experiment.

Before formal data collection, we conducted sample size calculations. First, according to G-Power software (Faul et al., 2007), considering an effect size (Cohen’s *d*) of 0.645 between two independent groups, a power of 0.8 and an*α* of 0.05, each group required 39 participants (*N* = 78). The effect size estimation was based on the lower limit confidence interval of the reported effect size for the CC effect in a previous study (Zhang et al., 2019).^1^ Next, researchers state that if we were to use the percentile bootstrap to examine the mediation effect, the minimum sample size to satisfy medium effect sizes for both the a-path and b-path of the mediating model, while considering a power of 0.8 and an *α* of 0.05, would be 78 participants (Fritz and MacKinnon, 2007). Consequently, the current sample size of 108 exceeded the minimum requirement to reach valid conclusions.

### All-or-none public goods game

The paradigm of the all-or-none public goods game was derived from Gill et al. (2013), and presented in the context of an environmental scenario (Pillutla and Chen, 1999; Zhang et al., 2019). Participants were asked to view themselves as corporate representatives attempting to build an environmental-protection-focused corporation with the cooperation of other group members.

At the onset of each round, every participant was given 50 tokens and was required to decide whether to donate all their tokens to an environmental group account (i.e., cooperative behavior) or contribute them to a personal account. The marginal *per capita* return (MPCR) for the group account is 0.6, which means each donation of 50 tokens into the group account results in a dividend of 30 tokens (i.e., 50 × 0.6) for each group member, including the contributor. Participants’ income for each round consisted of dividends from the group account and tokens in their personal account (see Table 1 for the payoff matrix). Participants were informed that everyone in the group would be randomly assigned an identity code, thus ensuring that the game would be played anonymously.

**Table 1:** Participants’ payoffs (tokens) matrix per round.

Participants’ decision	No others contribute	One other contributes	Two others contribute	Three others contribute
Contribute to group account	30	60	90	120
Contribute to personal account	50	80	110	140

### Measures

#### Moral elevation

Moral elevation was measured using a 9-item scale developed by Zhang et al. (2019) (see Supplementary Table S1). The scale was composed of three dimensions proposed by Aquino et al. (2011), namely emotional components (four items, sample item: “Please rate the level to which you felt moved after the public goods dilemma game”), views of humanity (three items, sample item: “Please rate the level to which you felt optimistic about humanity after the public goods dilemma game”), and desire to be a better person (two items, sample item: “I want to help others”). Participants answered each item on a 9-point Likert scale (1 = “did not feel at all,” 9 = “felt very strongly”). The overall Cronbach’s alpha was 0.91.

#### Calling

Calling was measured using five items adapted from Fry and Matherly (2006), revised to fit the public goods dilemma (see Supplementary Table S2). A sample item is as follows: “Contributing to the environmental group account is personally meaningful to me.” Participants answered each item on a 7-point Likert scale (1, strongly disagree; 7, strongly agree). Due to a technical error, we were not able to obtain the responses of 17 participants for the third item (i.e., “Contributing to the environmental group account is very important to me”), so we calculated the mean of their ratings for the other four items as the final mean value. The overall Cronbach’s alpha was 0.87.

#### Cooperative decision

Based on previous studies of CCs (Weber and Murnighan, 2008; Zhang et al., 2019), the total number of contributions to the group account in the last 10 rounds in both the first and second phases was defined as cooperative behavior. We excluded the decisions in the first five rounds from our analysis because, on average, it took the participants of a past research five rounds to get to know the new group members and to become aware of the presence of CCs (Zhang et al., 2019).

### Procedure

To better simulate real multi-person group decision-making, eight participants per game were invited to the laboratory. Upon arrival, each participant was seated in front of a shielded computer and provided informed consent documents to read and sign. They were then instructed to read the public goods game instructions and complete questions to verify that they understood the rules of the upcoming tasks.

The experiment was conducted on the z-Tree platform, a software that supports multi-person real-time decision-making interaction (Fischbacher, 2007). In the first phase of the experiment, participants were told that they were randomly assigned to an anonymous group of four, and were instructed to play 15 rounds of an all-or-none public goods game with three other group members. After 15 rounds, the participants completed a manipulation check question on a 7-point Likert scale (1, strongly disagree; 7, strongly agree). “There was someone in my group who always put their tokens in the group account” (Weber and Murnighan, 2008; Zhang et al., 2019). Subsequently, participants completed a questionnaire measuring moral elevation and calling.

Then, in the second phase, participants were notified that they were randomly assigned to a new group of four people, none of whom they had met before. This group was also informed to play 15 rounds of a public goods game. After the last round was completed, the participants were thanked for their time and dismissed.

### Statistical analyses

To test the different decisions in each round between the CC and control conditions, the present experiment used chi-square (crosstabs) tests. Independent t-tests were used to check the success of CC manipulation and to verify H1. Scholars noted that the Bayesian approach provides richer and more accurate information than classical inference using confidence intervals and *p* values (Kruschke, 2013; Wagenmakers et al., 2018; van de Schoot et al., 2021). As such, we also conducted a Bayesian independent t-test and reported the Bayes factor (BF_10_), indicating the likelihood for the data to support the alternative hypothesis over the null hypothesis in the model. A BF_10_ range between 1 and 3 indicated anecdotal evidence, a BF_10_ range between 3 and 10 indicated moderate evidence, and >10 indicated strong evidence for the presence of the effect under consideration, meanwhile, a range between 1/3–1 indicated anecdotal evidence, 1/10–1/3 indicated moderate evidence, and < 1/10 indicated strong evidence for the absence of the effect (Wetzels and Wagenmakers, 2012).

To verify H2 and H3, mediation analyses were performed using Model 4 in the PROCESS Marco of SPSS developed by Hayes (2013). To verify H4, a chain-mediation analysis was performed using Model 6 of PROCESS Macro. The bootstrapping method, with 5,000 samples, was used to quantify indirect effects, and 95% confidence intervals (CI) were generated for the best measure of the mediation effect and chain-mediation effect. If the CI contains zero, it indicates that there is no significant mediating effect at the 5% significance level.

The Bayesian analysis was carried out using JASP version 0.16.3 (JASP Team, 2022), and the rest of the analyses were performed using SPSS version 23.0 for Windows.

## Results

### Manipulation checks

In the first phase, the contribution rate of the CC group participants (72% from the 60 participants) in the first round was not significantly different from that of the control group participants (65% from the 48 participants, χ ^2^ = 0.620, *p* = 0.431, odds ratio (OR) = 1.387, 95%CI [0.614, 3.136]). This suggests that participants in the two conditions did not differ from the initial beliefs and expectations of the public goods game and their anonymous group members.

After the last round of the public goods game in the first phase, participants in the CC condition (*M* = 5.600, *SD* = 2.019) responded more positively to the question “There was someone in my group who always put their tokens in the group account” than did participants in the control condition (*M* = 3.583, *SD* = 2.172; *t* (106) = −4.988, *p* < 0.001, Cohen’s *d* = 0.966, BF_10_ = 6297.821). This demonstrates the effectiveness of CC manipulation.

### CC effect in the public goods game

**Figure 2:**
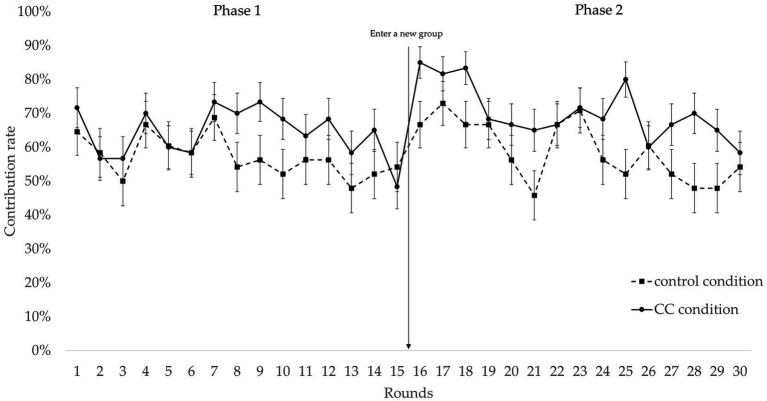
Participants’ rate of contribution to the group account for the two phases of the all-or-none public goods game.

Figure 2 presents the average contribution rate of participants in the 30 rounds of the public goods game.

The results showed that, in the first phase, participants in the CC group (*M* = 6.467, *SD* = 1.944) exhibited significantly more cooperative behavior than participants in the control group (*M* = 5.563, *SD* = 2.249; *t* (106) = −2.240, *p* = 0.027, Cohen’s *d* = 0.434, BF_10_ = 1.876). This again confirms the CC effect in the public goods dilemma.

### Hypothesis test

#### The contagion effect of CCs

In the second phase, we first compared participants’ contributions in the first round in both conditions. The results revealed that the CC group (85% from the 60 participants) contributed significantly more frequently than the control group (67% from the 48 participants, χ ^2^ = 5.038, *p* = 0.025, *OR* = 2.833, 95%CI [1.119, 7.171]). This suggests that participants in the CC group showed significantly more cooperative behavior than participants in the control groups when playing with the new team in the public goods game.

We then examined the difference in participants’ contribution rates in the two conditions in the last 10 rounds of the second phase. The results revealed that participants in the CC condition (*M* = 6.717, *SD* = 2.443) were significantly more cooperative in the new group than those in the control condition (*M* = 5.542, SD = 2.432; *t* (106) = −2.489, *p* = 0.014, Cohen’s *d* = 0.482, BF_10_ = 3.127), indicating moderate support for the CC contagion effect and H1.

#### The mediating effect of moral elevation

Table 2 presents the results of the mediation analyses, and Table 3 shows the results of the bootstrap tests.

**Table 2:** The results of the mediating effect of moral elevation and calling between the presence of a CC and participants’ cooperative decisions in the second phase.

Model	IV	*B*	SE	β	*t*	*p*	95% CI		Fit index		
							LLCI	ULCI	*R*	*R* ^2^	*F*
M1	DV: cooperative behaviors in the second phase										
	Constant	5.542	0.352		15.747	0.000	4.844	6.239	0.235	0.055	6.194^*^
	The presence of a CC	1.175	0.472	0.235	2.489	0.014	0.239	2.111			
M2	DV: elevation										
	Constant	4.729	0.202		23.397	0.000	4.328	5.130	0.205	0.042	4.664^*^
	The presence of a CC	0.586	0.271	0.205	2.160	0.033	0.048	1.123			
M3	DV: cooperative behaviors in the second phase										
	Constant	3.910	0.860		4.544	0.000	2.204	5.616	0.304	0.092	5.339^**^
	The presence of a CC	0.973	0.475	0.195	2.048	0.043	0.031	1.915			
	Moral elevation	0.345	0.167	0.197	2.072	0.041	0.015	0.675			
M4	DV: calling										
	Constant	5.416	0.141		38.447	0.000	5.136	5.695	0.204	0.042	4.597^*^
	The presence of a CC	0.405	0.189	0.204	2.144	0.034	0.031	0.780			
M5	DV: cooperative behaviors in the second phase										
	Constant	−0.150	1.240		−0.121	0.904	−2.609	2.309	0.472	0.222	15.015^***^
	The presence of a CC	0.749	0.440	0.150	1.704	0.091	−0.123	1.621			
	Calling	1.051	0.221	0.418	4.751	0.000	0.612	1.490			
M6	DV: calling										
	Constant	4.502	0.338		13.335	0.000	3.833	5.172	0.339	0.115	6.834^**^
	The presence of a CC	0.292	0.186	0.147	1.567	0.120	−0.078	0.662			
	Moral elevation	0.193	0.065	0.277	2.956	0.004	0.064	0.323			
M7	DV: cooperative behaviors in the second phase										
	Constant	−0.546	1.308		−0.418	0.677	−3.139	2.047	0.479	0.229	10.309^***^
	The presence of a CC	0.684	0.445	0.137	1.537	0.127	−0.199	1.566			
	Moral elevation	0.154	0.160	0.088	0.959	0.340	−0.164	0.472			
	Calling	0.990	0.230	0.393	4.298	0.000	0.533	1.447			

IV, independent variable; DV, dependent variable; B, unstandardized coefficient; SE, standard error; β, standardized coefficient; LLCI, lower limit confidence interval; ULCI, upper limit confidence interval.

* *p* < 0.05;

** *p* < 0.01;

*** *p* < 0.001.

**Table 3:** The chain-mediation effect and 95% confidence interval estimated by the bootstrap method.

Path		Effect	Standardized effect	SE	LLCI	ULCI
Indirect effect: condition→moral elevation→ cooperative behaviors		0.202	0.081	0.139	0.002	0.523
Indirect effect: condition→calling→ cooperative behaviors		0.426	0.171	0.268	0.028	1.058
Total indirect effect: condition→moral elevation→calling→cooperative behaviors		0.491	0.197	0.282	0.061	1.145
Indirect effect	Path 1: condition→moral elevation→ cooperative behaviors	0.090	0.018	0.113	−0.097	0.363
	Path 2: condition→calling→ cooperative behaviors	0.289	0.058	0.232	−0.058	0.836
	Path 3: condition→moral elevation→calling→cooperative behaviors	0.112	0.022	0.076	0.004	0.293

LLCI, lower limit confidence interval; ULCI, upper limit confidence interval.

**Figure 3:**
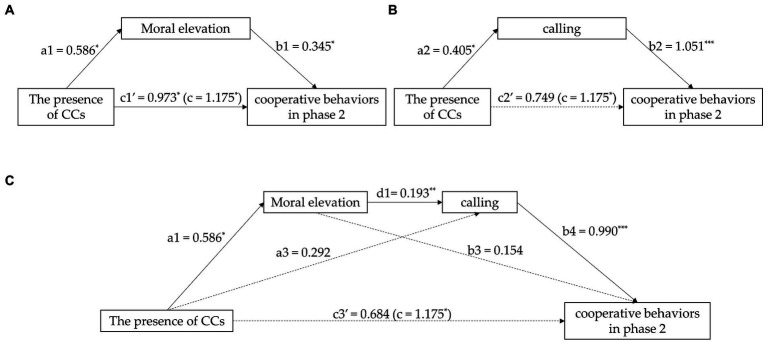
**(A)** The mediation effect of moral elevation in the relationship between the presence of CCs and cooperative behaviors in the second phase; **(B)** The mediation effect of calling in the relationship between the presence of CCs and cooperative behaviors in the second phase; **(C)** The chain-mediation effect of moral elevation and calling in the relationship between the presence of CCs and cooperative behaviors in the second phase. The path coefficients are unstandardized. Path coefficients with solid lines are significant; path coefficients with dashed lines are not significant; ^*^p < 0.05, ^**^p < 0.01, ^***^p < 0.001.

We examined the indirect effect of CCs on participants’ cooperative decisions *via* moral elevation (see Figure 3A). In Model 1, the presence of a CC significantly predicted individuals’ cooperative behavior in the second phase (*b* = 1.175, *SE* = 0.472, *p* = 0.014). In Model 2, the presence of a CC significantly predicted moral elevation (*b* = 0.586, *SE* = 0.271, *p* = 0.033). In Model 3, the presence of a CC and moral elevation significantly predicted individuals’ cooperative behavior in the second phase. Moreover, the result of the bootstrapping analysis revealed a significant mediating effect of 0.202, with a 95% CI of [0.002, 0.523], which did not contain zero, thus supporting H2.

#### The mediating effect of calling

We examined the indirect effect of the presence of a CC on participants’ cooperative behavior *via* calling (see Figure 3B). In Model 1, the presence of a CC significantly predicted individuals’ cooperative behavior in the second phase (*b* = 1.175, *SE* = 0.472, *p* = 0.014). In Model 4, the presence of a CC significantly predicted calling (*b* = 0.405, *SE* = 0.189, *p* = 0.034). In Model 5, the presence of a CC and calling significantly predicted individuals’ cooperative behaviors in the second phase. Moreover, the result of the bootstrapping analysis revealed a significant mediating effect of 0.426, with a 95% CI of [0.028, 1.058], which did not contain zero. Thus, H3 was supported.

#### The chain-mediating effect of moral elevation and calling

The bootstrap test showed that the total mediating effect of moral elevation and calling was significant, with a total indirect effect of 0.491 and 95% CI of [0.061, 1.145], which did not contain zero. In addition, the chain-mediating effect of moral elevation and calling (Path 3) was significant, with an effect of 0.112 and 95% CI of [0.004, 0.293], which did not contain zero, and which lends support for H4.

Combined with the hypotheses supported above, it can be demonstrated that the presence of a CC influenced individuals’ moral elevation and then influenced their callings, thus influencing their cooperative behaviors in the second phase. However, as shown in Figure 3C, the coefficient of the presence of a CC in Model 6 (*b* = 0.292, *SE* = 0.186, *p* = 0.120) and Model 7 (*b* = 0.684, *SE* = 0.445, *p* = 0.127) was not significant, and the coefficient of moral elevation in Model 7 was not significant (*b* = 0.154, *SE* = 0.160, *p* = 0.340). As we did not clarify whether we expect these paths to be present in the full chain mediation model, we evaluate the evidence in favor of H4 as moderate at best.

## Discussion

Plato has a classic parable that says “good actions give strength to ourselves and inspire good actions in others”(Capraro and Marcelletti, 2015). The contagion effect of helping implied by this statement has been validated in numerous studies (Tsvetkova and Macy, 2014; Chancellor et al., 2018; Norbutas and Corten, 2018; Jung et al., 2020). However, unlike helping role models, whose behaviors tend to be directed toward specific individuals, cooperative modeling behaviors are directed toward collectives without specific individual targets. As a result, it is still debatable whether cooperative models have the same contagion effect (Suri and Watts, 2011; Jordan et al., 2013).

Therefore, the main objective of this preliminary study was to examine the contagion effect of cooperative modeling and the underlying psychological mechanisms. Using a public goods game with an environmental framework, we discovered that the presence of a CC not only inspired group members to cooperate more inside the group but partially inspired them to more cooperative behavior in a subsequent game. Furthermore, drawing upon goal contagion theory, our findings highlighted the chain-mediating role of elevation and calling in the positive contagion effect of CC, thus supporting all our hypotheses. To the best of our knowledge, this study is the first to directly test the application of the two processes of goal contagion theory in a public goods game. We will start by discussing our findings using some specific theoretical perspectives.

First, the findings provide evidence for cooperative modeling literature by showing that the influence of cooperative role models on individuals’ immediate cooperative behaviors inside a group extends to the cooperative behaviors of these influenced individuals when they are outside the group. This study used an anonymous public goods game and had two phases between which group members were changed, which excluded the potential influence of homogeneity caused by fixed social networks. Despite this, we still discovered that CCs had a moderate impact, verifying H1. This finding is consistent with the results of previous research revealing that cooperation can spread from person A to person B to person C (Fowler and Christakis, 2010; Harrell, 2019; Jung et al., 2020).

In addition, the results suggest the explanation of goal contagion theory for the contagion effect of cooperative modeling. Specifically, previous research (e.g., CC effect-related studies) tended to focus on individuals’ imitative behaviors in the context of cooperative role models (Weber and Murnighan, 2008; Gill et al., 2013; Zhang et al., 2019) and various theories were devised to try and explain these behaviors, such as the logic appropriateness framework (Weber et al., 2004) and goal contagion theory (Jung et al., 2020). The former argues that individuals learn from CCs that cooperation is appropriate behavior in their setting and may be dependent on CCs, whereas the latter argues that individuals adopt pro-social goals independent of CCs. The results of our measurements for participants’ cooperative behaviors after they left the groups with CCs provide arguments to some extent for goal contagion theory.

Our research contributes to goal contagion theory by innovatively using moral elevation as a proxy variable of goal inference, and identifying this variable as a novel and important mediator. This theory posits that before an individual copies the cooperative model’s behaviors, the individual must first discern the model’s goals. Moral elevation is an emotional representation of an individual’s ability to infer and appraise others’ prosocial goals, and measuring it does not influence goal adoption, potentially addressing some of the obstacles previously faced in measuring explicit goal inference (Weber and Murnighan, 2008; Gill et al., 2013; Zhang et al., 2019). Previous research has discovered that moral elevation can motivate individuals to imitate CCs’ cooperative behaviors (Zhang et al., 2019). Our study extends this evidence by demonstrating that the impact of moral elevation stretches to individuals’ cooperative behaviors in a new group without CCs, and supporting the role of goal inference in the contagion effect of CC. While we caution that our result is a mere starting point for the application of goal contagion theory on cooperative behaviors, the use of moral elevation as an assessment of goal inference of others’ prosocial behaviors could prove promising in future studies.

Apart from the goal contagion theory, the reputation-management hypothesis could be a potential alternative explanation for the positive effect of moral elevation on the cooperative behaviors of CC group members in a new group. It describes that each individual actively shows others good qualities to develop and preserve their reputation, and then utilizes this reputation to obtain opportunities to align with others (Fessler and Haley, 2003; Radzvilavicius et al., 2019; Monroe, 2020). Individuals experience pressure when others improve their reputations through prosocial benevolence. Moral elevation is a cognitive strategy designed to deal with this pressure by eliciting object-indiscriminate benevolent behavior that enhances one’s reputation in all dimensions (Fessler and Haley, 2003). Thus, in response to witnessing CC’s cooperative behavior, members of the CC group might develop moral elevation to drive them to increase their object-indiscriminate cooperative behaviors to enhance their reputation. The fact that individuals from the CC groups showed more cooperative behaviors than in the control groups in both phases seems to provide evidence for the object-indiscriminate nature of benevolent behavior motivated by moral elevation.

Additionally, the current study underscores calling as another important mediator between the presence of a CC and individuals’ cooperative behaviors in new groups. One of the key aspects of the goal contagion theory is that individuals must adopt the goals of role models before they perform similar behaviors. We provide evidence of this by measuring individuals’ feelings of calling after playing 15 rounds of a public goods game with CCs. The findings indicated that the presence of a CC induces group members’ calling, a prosocial goal that drives them to exhibit cooperative behavior after their contact with CCs. Previous studies have found positive outcomes for calling, such as more green employee behavior, higher willingness to sacrifice for work, better work performance, and higher levels of life satisfaction (Hall and Chandler, 2005; Bunderson and Thompson, 2009; Allan and Duffy, 2014; Zhang et al., 2021). Our study extends calling-related literature to the field of cooperation in a social context.

Lastly, this study is the first to explicitly suggests the application of the two processes of goal contagion in the contagion effect of cooperative modeling. In light of the fact that directly assessing these two key components of goal contagion has proven a challenging endeavor in earlier investigations (Dik and Aarts, 2007; Corcoran et al., 2020), the current study extends the literature by newly suggesting the novel use of moral elevation and calling as proxy variables of goal inference and goal adoption, respectively. As presented in Figure 3, the findings revealed that the presence of CCs promoted moral elevation in group members, resulting in a sense of calling, which led to a high level of cooperative behavior in new/different groups even after leaving groups with CCs. This finding on the full chain-mediation partially helps explain the contagiousness of cooperative modeling *via* goal contagion theory.

### Implications

Although we are enthusiastic that the results of this preliminary study might have important implications for the “sharing economy,” “social media” or teams in large corporations (see details in the Supplementary Material S1), we want to discuss limitations and provide suggestions for future replication studies.

### Limitations and future study

Several limitations of this pre-study hinder us to draw stronger conclusions.

First, while novel, our measurement of moral elevation as a proxy for goal inference is limited to prosocial modeling contexts and thus may limit the generalizability of our results. This is because albeit moral elevation is a positive emotion that occurs after witnessing the prosocial behavior of others, goal contagion theory is not bounded to prosocial goals; instead, it encompasses a broader range of goals, such as dieting and achievement goals (Lee and Shapiro, 2016; King and Mendoza, 2020). Some researchers have attempted to employ implicit association tests; however, distinguishing between goal inference and goal adoption is challenging (Jia et al., 2014; Corcoran et al., 2020). Hence, future research should focus on developing appropriate assessment tools for goal inference.

Second, we used the public goods game paradigm in both phases to measure individuals’ cooperative behavior, hindering the exclusion of the possibility that individuals learned the norms of this paradigm. In other words, the cooperative behavior exhibited by individuals after leaving CCs may be related not only to the adoption of CCs’ prosocial goals but also to the fact that individuals learn from CCs that cooperation is the appropriate action in the paradigm of the public goods game. To prevent potential confounding, a different paradigm of cooperative behavior, such as common-pool resources, is recommended for future research.

Third, we set the average cooperation rate of the three computer-manipulated confederates (including the CC) in the control and CC groups at 66.7%, which limits the generalization of our results to a relatively cooperative group environment. There are several reasons why we set the percentage at 66.7%. First, we did not want the other two confederates to contribute so little that they became free riders, overshadowing the effect of the CC. Furthermore, previous similar studies discovered that people’s expectations of others’ cooperation rates in the first round were around 70%, as were their actual cooperation rates (Zhang et al., 2019). In line with this, the current study found an average cooperation rate of 69% in the first round. Therefore, we refer to the 66.7% cooperation rate set by Gill et al. (2013). However, we acknowledge that this is a relatively high number that shapes a relatively cooperative group norm for participants in both conditions, configuring a potential reason for participants in the CC group to have adopted prosocial goals. Follow-up studies can consider exploring the CC effect and its contagion in a group that is, on average, less pro-social, thus increasing the applicability of the relevant findings.

Fourth, our study revealed that cooperative behavior is contagious from A to B to C, but it did not consider the possibility that cooperative models inspire other prosocial behaviors in others. We discovered that cooperative models in public goods games have had an impact on participants’ cooperation rate in the same game and a subsequent game. Prosocial goals, such as a sense of calling, have been demonstrated to lead to a variety of positive consequences that are not restricted to cooperative behaviors (Bunderson and Thompson, 2009; Zhang et al., 2021). As a result, future research might consider the benefits of cooperative models on others’ prosocial actions outside of the group, such as helping and sharing behaviors.

Fifth, the 95% CIs for some of our results are wide, which may be due to the lack of a large sample. Although we cannot make up for this deficiency, we used the Bayesian approach in our data analysis to gain additional information about the probability that our hypotheses were supported, given the data (Wagenmakers et al., 2018). The results of the Bayesian t-test provide moderate evidence for the existence of the contagion effect of CC. However, the Bayesian approach does not completely compensate for the small sample, and hence future studies should replicate this study using a more conservative sample size estimating approach.

Sixth, the small to moderate effect sizes for all hypotheses may require replication attempts in future studies. Although the investigation of the CC contagion effect obtained moderate effect size, the effect sizes regarding the mediating effect and the chain mediation were relatively small. In particular, we only found a standardized effect size of 0.022 for the chain mediation effect of moral elevation and calling, with a lower limit confidence interval very close to 0, which indicates a lack of robustness. In this case, we cannot rule out the possibility of sampling error unless a replication study is conducted using a larger sample size. Therefore, the results require additional support from data before conclusions should be drawn. A potential setup for such a study is outlined in the Supplementary Material S2. Additionally, computational modeling has been proposed as a promising approach for advancing theories in psychological science (Guest and Martin, 2021; Robinaugh et al., 2021; Liu and Chen, 2022). Researchers suggest that it can ensure the quality, applicability, and authenticity of research by making the implicit model underlying the study explicit (Guest and Martin, 2021).

For future studies on this topic, we, therefore, suggest that researchers make use of our initial results and preregister a larger sample size for a potential replication study (see Supplementary Material S2) to meaningfully corroborate our findings. Computational modeling could additionally be used to clarify the theoretical considerations.

## Conclusion

In conclusion, this preliminary study discovered that cooperative modeling can not only inspire people’s immediate cooperative behavior within groups but also later cooperative behavior outside of these initial groups, without the presence of a role model. This study’s results potentially make some important contributions by suggesting the application of goal contagion theory to the CC contagion effect. Consistently experiencing the cooperative behaviors of CCs might inspire individuals’ moral elevation, which could lead to a sense of calling, inducing them to perform cooperative behaviors, regardless of the CC’s presence. The novel use of moral elevation and calling as proxies for goal inference and goal adoption, respectively, may provide researchers with new perspectives on assessing the two-stage process of goal contagion theory in prosocial circumstances. These findings, hence, bear the potential to enhance our knowledge of the cooperative modeling contagion effect, but given our small sample size, high-powered replication studies are necessary before stronger conclusions can be drawn. We hope that other scholars will be stimulated by this preliminary study to make greater progress in the field of cooperation.

## Data availability statement

The datasets presented in this study can be found in online repositories. The names of the repository/repositories and accession number(s) can be found at: https://osf.io/pvjkx/.

## Ethics statement

The studies involving human participants were reviewed and approved by Department of Psychology and Behavioral Sciences at Zhejiang University, China (2018-03-18). The patients/participants provided their written informed consent to participate in this study.

## Author contributions

QZ contributed to the study design, data collection, data analysis, interpretation of results, and writing and critical review of the manuscript. JM contributed to the study design, data collection, interpretation of results, and review of the manuscript. YW contributed to data analysis, the interpretation of results, and review of the manuscript. XL contributed to the review of the manuscript. CF contributed to the data collection and review of the manuscript. All authors have read and agreed to the published version of the manuscript.

## Funding

This research was funded by National Natural Science Foundation of China, grant numbers 72101232 and 71871201, and Natural Science Foundation of Zhejiang Province, grant number LQ22G010008. The APC was funded by National Natural Science Foundation of China, grant number 72101232.

## Conflict of interest

The authors declare that the research was conducted in the absence of any commercial or financial relationships that could be construed as a potential conflict of interest.

## Publisher’s note

All claims expressed in this article are solely those of the authors and do not necessarily represent those of their affiliated organizations, or those of the publisher, the editors and the reviewers. Any product that may be evaluated in this article, or claim that may be made by its manufacturer, is not guaranteed or endorsed by the publisher.

## Footnotes

^1^To calculate the effect sizes, we obtained the following data from that study (Zhang et al., 2019): For the CC condition, *n* = 100, *M* = 6.63, *SD* = 2.64, and for the control condition, *n* = 96, *M* = 4.22, *SD* = 2.48. We were able to calculate Cohen’s *d* = 0.94 with 95%CI [0.645, 1.235].
