# Supplementary material for: Ripples in the pond: Evidence for contagious cooperative role modeling through moral elevation and calling in a small pre-study
Source: Front Psychol. 2022 Oct 20;13:1005772. doi: 10.3389/fpsyg.2022.1005772 (PMC9630473; doi:10.3389/fpsyg.2022.1005772)
Supplement: Supplementary file 1 [file Data_Sheet_1.docx]

Supplementary Material

**Table S1.** The scale of moral elevation

| dimension | items | M | SD |
| --- | --- | --- | --- |
| emotional components | 1. Please rate the level to which you felt *moved* after the public goods dilemma game | 4.176 | 2.117 |
|  | 2. Please rate the level to which you felt *uplifted* after the public goods dilemma game | 4.324 | 1.833 |
|  | 3. Please rate the level to which you felt *admiration* after the public goods dilemma game | 4.787 | 2.171 |
|  | 4. Please rate the level to which you felt *warm feeling in chest* after the public goods dilemma game | 4.148 | 1.947 |
| views of humanity | 5. Please rate the level to which you felt *optimistic about humanity* after the public goods dilemma game | 4.870 | 1.757 |
|  | 6. Please rate the level to which you felt that *there is still some good in the world* after the public goods dilemma game | 5.796 | 1.863 |
|  | 7. Please rate the level to which you felt that *People are really good* after the public goods dilemma game | 5.417 | 1.762 |
| desire to be a better person | 8. I want to help others | 5.731 | 1.694 |
|  | 9. I want to become a better person | 6.241 | 1.522 |

Note: *N* = 108.

**Table S2.** The scale of calling

| items | M | SD |
| --- | --- | --- |
| 1.The environmental group account is meaningful to me. | 5.519 | 1.196 |
| 2.Contributing to the environmental group account is personally meaningful to me. | 5.704 | 1.284 |
| 3.Contributing to the environmental group account is very important to me. | 5.846 | 1.105 |
| 4.My contribution to the environmental group account makes a difference in people’s lives. | 5.907 | 1.115 |
| 5.I believe that contributing to the environmental group account is for the benefit of everyone, not for my own benefit. | 5.343 | 1.334 |

Note: *N* = 108; the third item has 17 missing value (*n* = 91).

# S1 Potential Implications

These results have several important implications.

First, practitioners in industries that value mutual cooperation among individuals, corporations, and even governments may consider encouraging consistent cooperative behaviors among small groups. For example, the “sharing economy” has recently received considerable attention because of its low-carbon enhancement (van Dillen et al., 2021). The encouragement of a group of cooperative models aids in the development of cooperation, producing an ecology of goodwill circulation that can significantly improve the efficiency of the use of goods or services.

In addition, practitioners can benefit from this study’s findings by gaining insight into how cooperative models transform others into cooperative models in new/different settings. Cooperative models can be encouraged to share their prosocial goals to reduce the cost of goal inference for others and assist others in quickly adopting and implementing prosocial goals. With the spread and the growth of social media as an important communication tool for information generation and dissemination in the post-pandemic era, such sharing may impact a large number of people, just like ripples radiating from the dropping of a stone into a pool.

# S2 Research Proposal for study 2

This is a hypothetical protocol for study 2, which should be conducted to confirm the results from the pre-study in the paper. This protocol can be a blueprint for a future registered report protocol.

# Introduction

In the previous study, we discovered that those who encountered CC demonstrated more cooperative behavior when they entered new groups than people in the control group. Using goal contagion theory to explain this phenomenon, we investigated the proxy variables moral elevation for goal inference and calling for goal adoption. Based on the findings, moral elevation and calling have a chain-mediating influence on the relationship between the presence of CCs and individuals' cooperative decisions after switching to a new group.

Although we uncovered evidence to support goal contagion theory using proxy variables, we cannot rule out the possibility that people' high cooperative actions after leaving CC were due to role modeling or behavioral mimicry (Chartrand and Lakin, 2013; Morgenroth et al., 2015; Jung et al., 2020). The primary distinction between goal contagion theory and role modeling or mimicry is that when an individual adopts the same goal as the role model, he or she can put the goal into action by engaging in different behaviors (Brohmer et al., 2019).

As a result, the primary purpose of this study was to determine whether individuals were capable of exhibiting higher levels of prosocial behaviors other than cooperation after being exposed to CC when compared to persons in the control group. We chose sharing behaviors among the numerous prosocial behaviors because it is a classic altruistic behavior that has been widely examined by a large number of researchers and has a well-established measurement paradigm, the dictator game (Bardsley, 2008; Edele et al., 2013; van Dillen et al., 2021). Furthermore, we expect to use the two proxy variables, moral elevation and calling, to validate the two-stage process of goal contagion theory under such circumstance.

## The current study

In light of the above arguments, we then propose the following hypothesis (see Table S3).

H1: Participants in the CC group will exhibit sharing behaviors to strangers than those in the control group after leaving their groups.

H2: Moral elevation mediates the influence of CCs on participants’ sharing behaviors after participants enter a new group.

H3: Calling mediates the influence of CCs on participants’ sharing behaviors after participants enter a new group.

H4: Moral elevation and calling have a chain-mediating effect on the relationship between CCs and participants’ subsequent pro-environmental decisions after participants shift to a different context without CCs.

# Methods

## Design and procedure

The study will have two conditions. Participants will be allocated to either the CC condition or the control condition at random. Each condition consists of two phases.

In the first phase, participants are formed into groups of four and had to play 15 rounds of an "all-or-none" public goods game. To control for the effects of social norms on individuals’ cooperative behavior (Farrow et al., 2017), the three other group members (including CC) who interact with participants in both phases are computer-manipulated confederates with an average 66.7% likelihood of cooperative behavior (Gill et al., 2013). The CC condition differs from the control condition is that one of the simulated group members in the CC condition is a CC who will consistently make cooperative decisions (i.e., contributing all the tokens to the group account).

After 15 rounds of public goods game, the participants respond to a manipulation check question. Following that, participants complete a questionnaire to assess moral elevation and calling.

The second phase is the same for both conditions. Participants are instructed to play one round of the dictator game as the proposer with a stranger they have never encountered before.

## Measure

### Moral Elevation

We use a 9-item scale developed by Zhang et al. (2019) to measure moral elevation (see Table S1). The scale is composed of three dimensions proposed by Aquino et al. (2011), namely emotional components (four items, sample item: “Please rate the level to which you felt moved after the public goods dilemma game”), views of humanity (three items, sample item: “Please rate the level to which you felt optimistic about humanity after the public goods dilemma game”), and desire to be a better person (two items, sample item: “I want to help others”). Participants response to each item on a 9-point Likert scale (1 = “did not feel at all,” 9 = “felt very strongly”).

### Calling

We use a 5-item scale adapted from Fry and Matherly (2006) and revised to fit the public goods dilemma to measure calling (see Table S2). A sample item is as follows: “Contributing to the environmental group account is personally meaningful to me.” Participants response to each item on a 7-point Likert scale (1 = “strongly disagree,” 7 = “strongly agree”).

### "All-or-none" Public Goods Game and Cooperative Behavior

The paradigm of the “all-or-none” public goods game is derived from Gill et al. (2013), and presented in the context of an environmental scenario (Pillutla and Chen, 1999; Zhang et al., 2019). Participants are invited to imagine themselves as corporate representatives attempting to develop a corporation focused on environmental protection with the cooperation of other group members.

Each participant is given 50 tokens at the start of each round and is required to choose whether to donate all of their tokens to an environmental group account (i.e., cooperative behavior) or to a personal account. The group account's marginal per capita return (MPCR) is 0.6, which indicates that a donation of 50 tokens results in a dividend of 30 tokens (i.e., 50 0.6) for each group member, including the contributor. Dividends from the group account and tokens in their personal account comprise each round's income for participants (see Table S4 for the payoff matrix). Everyone in the group was informed that they would be randomly allocated an identity code, assuring that the game would be played anonymously.

Based on previous studies of CCs (Weber and Murnighan, 2008; Zhang et al., 2019), the total number of contributions to the group account in the last 10 rounds in the first phase is defined as cooperative behavior. We exclude participants' decisions in the first five rounds from the analysis because it takes them on average five rounds to get to know the new group members and to become aware of the presence of CCs (Zhang et al., 2019).

### Sharing Behavior

Sharing behavior is measured using the dictator game (Bardsley, 2008; Edele et al., 2013; van Dillen et al., 2021). The dictator game consists of a proposer and a responder. The proposer receives 100 tokens at the beginning of the game and needs to decide on how to allocate these tokens between the two players. The responder has no choice but to accept whatever number of tokens the proposer proposes to share with him/her. The participants will play the dictator game only once as the proposer. The dependent variable was the number of tokens participants decided to allocate to the responder.

### Manipulation check

Participants need to respond to the manipulation check question on a 7-point Likert scale (1 = “strongly disagree,” 7 = “strongly agree”): “There was someone in my group who always put their tokens in the group account” (Weber and Murnighan, 2008; Zhang et al., 2019).

We believe that if the manipulation checks fail but hypotheses are nevertheless supported, this could be due to participants’ implicitly being influenced by CCs without realizing their existence.

## Sample

Based on the Monte-Carlo simulations, Schönbrodt and Perugini (2013) proposed that the required effect size for a stable estimate is N = 250. Using the smallest effect size of interest (SESOI) and the TOSTER package in R, we calculate that if we plan to recruit a sample size of 250 participants, the Cohen’s *d* has to reach 0.37 in order to reach alpha = 0.05, power = 0.80. Therefore, we plan to recruit 250 participants and set the effect size to be meaningful only if it reaches 0.37.

# Reference

Aquino, K., McFerran, B., and Laven, M. (2011). Moral identity and the experience of moral elevation in response to acts of uncommon goodness. *J. Pers. Soc. Psychol.* 100, 703–718. doi: 10.1037/a0022540.

Bardsley, N. (2008). Dictator game giving: altruism or artefact? *Exp. Econ.* 11, 122–133. doi: 10.1007/s10683-007-9172-2.

Brohmer, H., Fauler, A., Floto, C., Athenstaedt, U., Kedia, G., Eckerstorfer, L. V., et al. (2019). Inspired to Lend a Hand? Attempts to Elicit Prosocial Behavior Through Goal Contagion. *Front. Psychol.* 10, 545. doi: 10.3389/fpsyg.2019.00545.

Chartrand, T. L., and Lakin, J. L. (2013). The Antecedents and Consequences of Human Behavioral Mimicry. *Annu. Rev. Psychol.* 64, 285–308. doi: 10.1146/annurev-psych-113011-143754.

Edele, A., Dziobek, I., and Keller, M. (2013). Explaining altruistic sharing in the dictator game: The role of affective empathy, cognitive empathy, and justice sensitivity. *Learn. Individ. Differ.* 24, 96–102. doi: 10.1016/j.lindif.2012.12.020.

Farrow, K., Grolleau, G., and Ibanez, L. (2017). Social Norms and Pro-environmental Behavior: A Review of the Evidence. *Ecol. Econ.* 140, 1–13. doi: 10.1016/j.ecolecon.2017.04.017.

Fry, L. W., and Matherly, L. L. (2006). Spiritual leadership and organizational performance: An exploratory study. in (Atlanta, Georgia).

Gill, M. J., Packer, D. J., and Van Bavel, J. (2013). More to morality than mutualism: Consistent contributors exist and they can inspire costly generosity in others. *Behav. Brain Sci.* 36, 90–90. doi: 10.1017/s0140525x12000799.

Jung, H., Seo, E., Han, E., Henderson, M. D., and Patall, E. A. (2020). Prosocial modeling: A meta-analytic review and synthesis. *Psychol. Bull.* 146, 635–663. doi: 10.1037/bul0000235.

Morgenroth, T., Ryan, M. K., and Peters, K. (2015). The Motivational Theory of Role Modeling: How Role Models Influence Role Aspirants’ Goals. *Rev. Gen. Psychol.* 19, 465–483. doi: 10.1037/gpr0000059.

Pillutla, M. M., and Chen, X.-P. (1999). Social Norms and Cooperation in Social Dilemmas: The Effects of Context and Feedback. *Organ. Behav. Hum. Decis. Process.* 78, 81–103. doi: 10.1006/obhd.1999.2825.

Schönbrodt, F. D., and Perugini, M. (2013). At what sample size do correlations stabilize? *J. Res. Personal.* 47, 609–612. doi: 10.1016/j.jrp.2013.05.009.

van Dillen, L., Lelieveld, G.-J., Hofmann, W., and de Kwaadsteniet, E. W. (2021). ‘Sharing in need’: How allocator and recipient’s hunger shape food distributions in a dictator game. *J. Exp. Soc. Psychol.* 95, 104152. doi: 10.1016/j.jesp.2021.104152.

Weber, J. M., and Murnighan, J. K. (2008). Suckers or saviors? Consistent contributors in social dilemmas. *J. Pers. Soc. Psychol.* 95, 1340–1353. doi: 10.1037/a0012454.

Zhang, Q., Chen, Y., Tao, Y., Farid, T., and Ma, J. (2019). How Consistent Contributors Inspire Individuals to Cooperate: The Role of Moral Elevation and Social Value Orientation. *Sustainability* 11, 1874. doi: 10.3390/su11071874.

**Table S3.** Hypotheses and main variables.

| Hypothesis | | IV | mediator | DV |
| --- | --- | --- | --- | --- |
| H1 | Participants in the CC group will exhibit sharing behaviors to strangers than those in the control group after leaving their groups. | Condition: CC vs control | / | Sharing behavior |
| H2 | Moral elevation mediates the influence of CCs on participants’ sharing behaviors after participants enter a new group. |  | Moral elevation |  |
| H3 | Calling mediates the influence of CCs on participants’ sharing behaviors after participants enter a new group. |  | calling |  |
| H4 | Moral elevation and calling have a chain-mediating effect on the relationship between CCs and participants’ subsequent pro-environmental decisions after participants shift to a different context without CCs. |  | Moral elevation and calling |  |

*Note*: IV = independent variable; DV = dependent variable.

**Table S4.** Participants’ payoffs (tokens) matrix per round.

| **Participants’ decision** | **No others contribute** | **One other contributes** | **Two others contribute** | **Three others contribute** |
| --- | --- | --- | --- | --- |
| Contribute to group account | 30 | 60 | 90 | 120 |
| Contribute to personal account | 50 | 80 | 110 | 140 |
